# Electromagnetic wave transparency of X mode in strongly magnetized plasma

**DOI:** 10.1038/s41598-021-94029-3

**Published:** 2021-07-21

**Authors:** Devshree Mandal, Ayushi Vashistha, Amita Das

**Affiliations:** 1 Institute for Plasma Research, HBNI, Bhat, Gandhinagar, 382428 India; 2 Homi Bhabha National Institute, Mumbai, 400094 India; 3 Department of Physics, Indian Institute of Technology Delhi, Hauz Khas, New Delhi, 110016 India

**Keywords:** Physics, Plasma physics

## Abstract

An electromagnetic (EM) pulse falling on a plasma medium from vacuum can either reflect, get absorbed or propagate inside the plasma depending on whether it is overdense or underdense. In a magnetized plasma, however, there are usually several pass and stop bands for the EM wave depending on the orientation of the magnetic field with respect to the propagation direction. The EM wave while propagating in a plasma can also excite electrostatic disturbances in the plasma. In this work Particle-In-Cell simulations have been carried out to illustrate the complete transparency of the EM wave propagation inside a strongly magnetized plasma. The external magnetic field is chosen to be perpendicular to both the wave propagation direction and the electric field of the EM wave, which is the X mode configuration. Despite the presence of charged electron and ion species the plasma medium behaves like a vacuum. The observation is understood with the help of particle drifts. It is shown that though the two particle species move under the influence of EM fields their motion does not lead to any charge or current source to alter the dispersion relation of the EM wave propagating in the medium. Furthermore, it is also shown that the stop band for EM wave in this regime shrinks to a zero width as both the resonance and cut-off points approach each other. Thus, transparency to the EM radiation in such a strongly magnetized case appears to be a norm.

## Introduction

A wide range of intense magnetic fields exist in astrophysical (e.g. in galaxy clusters, shock formation in gamma ray bursts, magnetosphere of neutron stars^3–6^) as well as in the laboratory plasmas^7^. On one hand there has been several ongoing research to generate intense magnetic fields in the laboratory, while on the other, several instabilities lead to the generation of intense magnetic field in cosmological space (e.g. magnetic field generation via Kelvin-Helmholtz instability, counter-streaming electron flows etc^8–10^). These magnetic fields can be very strong, for instance, near pulsars and magnetars they could be of the order of Giga Teslas^11–13^ and a record magnetic field of 1.2 KT has been achieved in the laboratory^7^. It is thus important to study interaction of an electromagnetic wave with a strongly magnetized plasma for both laboratory as well as astrophysical context^1,2^ ^14–16^. The principle mechanisms in plasmas depend on EM wave frequency and plasma permittivity. Plasma permittivity can be altered by suitably choosing the plasma density and applied magnetic field. The free charges and their associated currents in the plasma medium act as sources and influence the plasma dielectric constant. Propagation of EM wave through strongly magnetized plasma sources, therefore, needs to be understood.

The issue of EM wave achieving complete transparency is important and has been considered earlier in many contexts. An attempt to achieve transparency using strong fields generated by intense femto-second (fs) laser pulse has been studied by^17–21^. Total transmission was observed when 30 fs laser pulse of intensity $3\times 10^{18}$ W $\text{cm}^{-2}$ passes through $0.1 \; \upmu$m plastic foil targets^18^. This mechanism is operative when the target width is much smaller than the laser wavelength^17^. However, for thick targets, relativistic laser would lead to excitation of coherent structures and/or instabilities leading to turbulence in the system^22,23^.

External magnetic fields have been applied in several contexts to achieve transparency. The dressing of resonance states for RHCP (Right Hand Circularly Polarized) waves with the combination of axial and wiggler magnetic field is attempted to acquire a window of transparency in the opaque magnetized plasma for the EM wave^24^. Another technique to seek transparency is by employing pump electromagnetic wave to transmit the probe wave^25–28^. This method is analogous to a quantum mechanical phenomena known as EIT (Electromagnetically Induced Transparency). In this phenomena, an electromagnetic wave is made to propagate in normally opaque medium in presence of powerful secondary EM waves. This is possible due to the destructive interference between several energy levels connecting the ground and excited states of the atom. This method is heavily used in non-linear optics to manipulate the energy levels of atomic states or slow down the waves^29–31^. In plasma, the use of pump and probe EM wave is used to make plasma transparent to RHCP wave. But this study is limited to propagation of RHCP waves along the magnetic field lines and generating an additional wiggler magnetic field is a complication from application viewpoint. More importantly these methods focus on R-wave mode where propagation is along the magnetic field, while L-wave and X-mode propagation geometries are yet to be explored.

**Figure 1:**
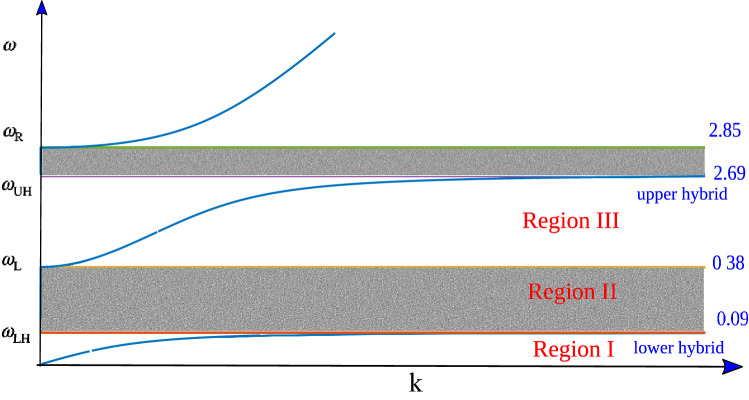
Dispersion of X-mode when ${\vec {k}} \perp {\vec{B_0}}$, classified into three regions. The numbers on the right hand side correspond to resonance and cutoff frequencies for $m_i=100m_e$ in normalized units.

We have carried out Particle-In-Cell simulations to study the propagation of EM wave in a strongly magnetized plasma for which both the electron and ion species are strongly magnetized. An interesting observation of complete transparency of the plasma medium is observed for the propagation of EM wave in the X mode configuration. The width of the stop band of the X mode reduces to zero and a completely transparent propagation of EM wave is observed. For X-mode, dispersion curve is given in Fig. 1, there are two stop bands ($\omega _{LH}-\omega _{L}$ and $\omega _{UH}-\omega _{R}$, where *LH*, *UH*, *L*, *R* stands for Lower Hybrid, Upper Hybrid, Left Hand Cutoff, Right Hand Cutoff respectively). Whenever laser/EM wave frequency lying on these bands is incident on plasma, it generates a shielding electric field in response to avoid penetration of EM wave inside it. The propagation of EM wave in plasma thus depends on the intensity and/or frequency of the incident EM wave. We show that when the strong external magnetic field dominates the motion of both charged species, i.e. $\omega _{ce}> \omega _{ci} > \omega _{l}$, or it strongly magnetizes electrons but the perturbations are at faster time scales than that of ions $\omega _{ce}> \omega _{l} > \omega _{L}$, the electromagnetic wave propagates undisturbed inside plasma. The inequalities gives an insight to plasma in terms of anisotropy and modification in its collective behaviour that gets affected by introduction of magnetic field. Such caveats are vital for absorption phenomena as well.

A strong magnetic field in which the two charged species remain closely tied to the magnetic field at the EM wave frequency, ensures that they do not provide the plasma a chance to respond to the incoming laser/EM wave. We show that as the strength of magnetic field increases, the magnitude of self generated fields in bulk plasma due to interaction of laser with plasma decreases and the EM wave propagation speed inside the plasma approaches the speed of light *c*. The plasma medium thus acts transparent to the incoming EM wave. These observations would have important implications in astrophysics. For instance, the strength of magnetic field in neutron stars and magnetars are very high of the order of Giga-Tesla. Plasma medium near these astrophysical objects would easily be in strongly magnetized regime for our results to be applicable for visible and even higher frequency radiations.

## Simulation details

**Figure 2:**
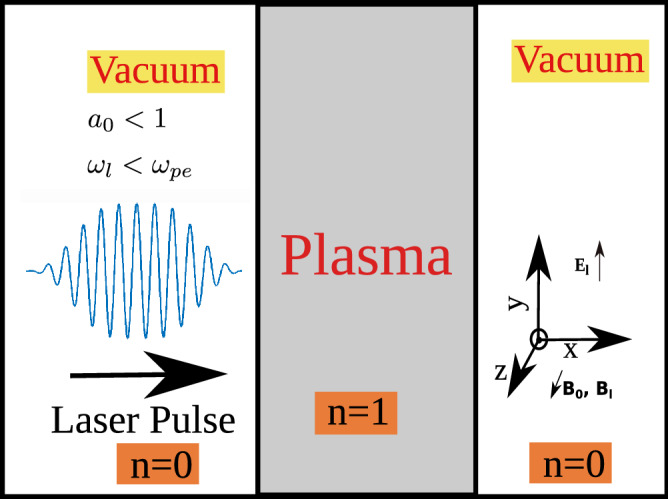
Schematic of simulation geometry [not to scale]. Plasma slab is placed in vacuum, direction of external Magnetic field ($B_0$) is along the same direction as that of the magnetic field of laser ($B_l$) i.e. $\hat{z}$. Electric field of laser is along $\hat{y}$ direction. $a_0$ is normalized vector potential i.e. ($eE_0/m\omega c$). This parameter decides the regime of plasma dynamics, in this work we have taken $a_0<1$ i.e. non-relativistic regime. In this schematic, ‘n’ denotes plasma density.

We have carried out series of one dimensional (along $\hat{x}$) Particle In Cell(PIC) simulations in X-mode configurations using OSIRIS-4.0^32–34^. For X-mode configuration, uniform external magnetic field ($B_0(m_e\omega _{pe}c/e)$) has been applied in $\hat{z}$ direction. A uniform plasma density comprising of electrons and ions has been considered. Ion mass is taken to be 100 times mass of electrons ($m_i=100m_e$) for faster computation. Plasma boundary extends from $x=850 c/\omega _{pe}$ to $x=2000c/\omega _{pe}$ whereas total length of simulation box is $3000 c/\omega _{pe}$. Boundary condition for particles as well as fields are absorbing, with spatial and temporal grid taken to be 0.05 and 0.02 respectively. A p-polarized, plane laser pulse is incident normally at plasma ($n=3.14 \times 10^{20}$ c.c) from the left boundary. These values of plasma density will differ with EM wave frequency for different region of X-mode according to ratio given in Table 1. Laser is propagating along $\hat{x}$ with its spatial profile centered at $x= 450 c/\omega _{pe}$ and ranging from $x= 0$ to $800 c/\omega _{pe}$. We also want to clarify that this work focuses on proof of concept so the mechanism presented in this paper depends on the magnetization of the charge species with respect to the incoming EM pulse frequency. We have carried out a parametric study on magnetic fields such that broadly they follow either criteria I ($\omega _{ce}> \omega _{l} > \omega _{ci}$) or criteria II ($\omega _{ce}> \omega _{ci} > \omega _{l}$). This parametric study has been done with laser pulse of intensity lying in non-relativistic regime such that amplitude of laser electric field ($E_0=0.03(m_e \omega _{pe}c/e)$) is constant for all runs. This has been done to avoid other relativistic mechanism to play a role. A schematic of simulation geometry has been shown in Fig. 2. Different laser frequency maintaining criteria I and criteria II has also been chosen according to frequency which is explained more elaborately in next section. A tabular form of simulation parameters is given in Table 1.

**Table 1:** Details of various simulation runs used in this study based on different regions of X-mode shown in Fig. 1.

	$B_0$ criteria I ($\omega _{ce}> \omega _{l}>\omega _{ci}$)	$B_0$ criteria II ($\omega _{ce}>\omega _{ci}>\omega _{l}$)	$\omega _l$ (in $\omega _{pe}$)	$a_0(\frac{eE_0}{m\omega c})$	$E_0$
Region I (0-$\omega _{LH}$)	3, 8	20	0.05	0.6	0.03
Region II ($\omega _{LH}-\omega _{L}$)	3, 8	20, 40	0.2	0.15	0.03
Region III ($\omega _{L}-\omega _{UH}$)	(0.25, 0.5 $\omega _{ce}<\omega _l$), 3, 8	40	0.5	0.06	0.03

## Results

### Theory and analytical assessment

It is well known that when EM wave is propagating perpendicular to external magnetic field, plasma supports two kinds of waves, O-mode (ordinary wave) and extraordinary mode (X-mode). O-mode is independent of applied magnetic field (ordinary wave). The general dispersion relation for perpendicular propagation in cold plasma ($\mathbf {k}\perp \mathbf {B}$) is given by the matrix^35^,

1 $$\begin{aligned} \begin{bmatrix} S &{} -iD &{} 0\\ iD &{} S-n^2 &{} 0\\ 0 &{}0 &{} P-n^2 \end{bmatrix} \begin{bmatrix} E_x\\ E_y\\ E_z \end{bmatrix}=0 \end{aligned}$$

where, $$S=\frac{1}{2}\left( R+L\right)$$, $$D=\frac{1}{2}\left( R-L\right)$$, $$P=1-\frac{\omega ^2_{p}}{\omega ^2}$$

2 $$\begin{aligned}&R=1- \frac{\omega _{pe}^2 + \omega _{pi}^2}{(\omega + \omega _{ci})(\omega - \omega _{ce})} \end{aligned}$$

3 $$\begin{aligned}&L=1- \frac{\omega _{pe}^2 + \omega _{pi}^2}{(\omega - \omega _{ci})(\omega + \omega _{ce})} \end{aligned}$$

The X-mode has cut-offs at $\omega _R$ and $\omega _L$ respectively. $\omega _R$ and $\omega _L$ are given as follows:

4 $$\begin{aligned} \omega _{R,L}= [\omega _{pe}^2+\omega _{pi}^2+(\omega _{ci}+\omega _{ce})^2/4]^{1/2} \mp (\omega _{ci}-\omega _{ce})/2 \end{aligned}$$

Dispersion curve of X-mode is shown in Fig. 1. We have  labelled dispersion curve into three regions depending on the dominant role played by the species. Region I is dominated by dynamics of ions and Region III by electrons. Region II is stop band as it lies between $\omega _{LH}$ (resonance point) and $\omega _{L}$ (cut-off point).

Dispersion relation for X-mode is obtained,

5 $$\begin{aligned} n^2= \frac{RL}{S} \end{aligned}$$

where, *n* is refractive index. Resonance occur when $S \rightarrow 0$

6 $$\begin{aligned} \omega ^4-\left( \omega _{pe}^2+\omega _{pi}^2+\omega _{ce}^2+\omega _{ci}^2\right) \omega ^2+\omega _{ci}^2\omega _{ce}^2+\omega _{pe}^2\omega _{ci}^2+\omega _{pi}^2\omega _{ce}^2=0 \end{aligned}$$

This is a bi-quadratic equation, it’s lower end solution is plotted as function of applied magnetic field in Fig. 4. As can be seen from the figure, when $B_0<10$ (at $B_0=10, \omega _{ci}=\omega _{pi}$) it falls in criteria I and solution of Eq. (6) matches perfectly with reduced expression of $\omega _{LH}$. At higher magnetic fields, $\omega _{LH}$ saturates at $\omega _{pi}$ while solution of Eq. (6) approaches left hand cut off ($\omega _{L}$) asymptotically which concludes that at this higher magnetic field the resonance point and cut off approach each other thus effectively reduce the width of the stop band. This was checked by simulation as well for frequency parameter lying in region II (i.e. stop band). Under criteria I, laser reflected back. On the other hand, under criteria II laser pulse was able to propagate through the plasma. This was possible due to effective reduction of stop band and resonance point lying well above EM frequency. So, effectively this case does not lie in region II but in region I.

**Figure 3:**
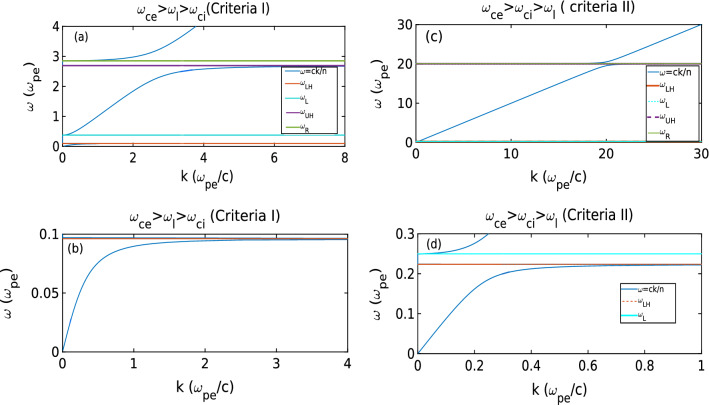
Dispersion curve for two criteria [criteria I $(\omega _{ce}>\omega _l>\omega _{ci})$ and criteria II ($\omega _{ce}>\omega _{ci}>\omega _{l}$) are shown in subplot (**a**) and (**c**) respectively]. In subplot (**b**) and (**d**), we expand the dispersion curves shown in (**a**) and (**c**) respectively. From these curves, we can observe that while criteria I has two stop bands and three pass bands, criteria II dispersion has $\omega =ck$ all along.

In Fig. 3, we have plotted the dispersion curve for X-mode in two criteria. As one can observe from the left subplot that in criteria I, all the regions are well separated while in the criteria II stop band has shrunk. Moreover, the dispersion follows $\omega =k$ (as $c=1$). Therefore, it rules out any other mode excitation when $\omega _{ce}> \omega _{ci} > \omega _{l}$.

To summarize our propagation characteristics according to their region of dispersion curve in Fig. 1 is given in Table 2.

**Table 2:** Details of propagation characteristics observed in various simulation runs for different regions of dispersion curve.

	Criteria I	Criteria II
Region I	LH	Transparent
Region II	Stop band	Transparent
Region III	Transparent	N.A

Detail quantitative analysis to calculate absorption (A), reflection (R), transmission (T) coefficients has also been done which is presented here in tabulated form (Table 3).

**Table 3:** A comparison of various coefficients with different external magnetic field.

	$B_0$	R	T	A
Region I	3	0.67	8.9 × $10^{-9}$	0.32
	8	0.0663	0.872	0.0617
	20	0.0035	0.9931	0.0034
Region II	3	0.99	7.95 × $10^{-9}$	1.9 × $10^{-5}$
	8	0.998	$8\times 10^{-7}$	0.00199
	20	0.0089	0.9823	0.0088
	40	5.6 × $10^{-4}$	0.9989	0.00054
Region III	3	0.013	0.9742	0.0128
	8	0.0006	0.9988	0.0006
	40	0.0035	0.9931	0.0034

**Figure 4:**
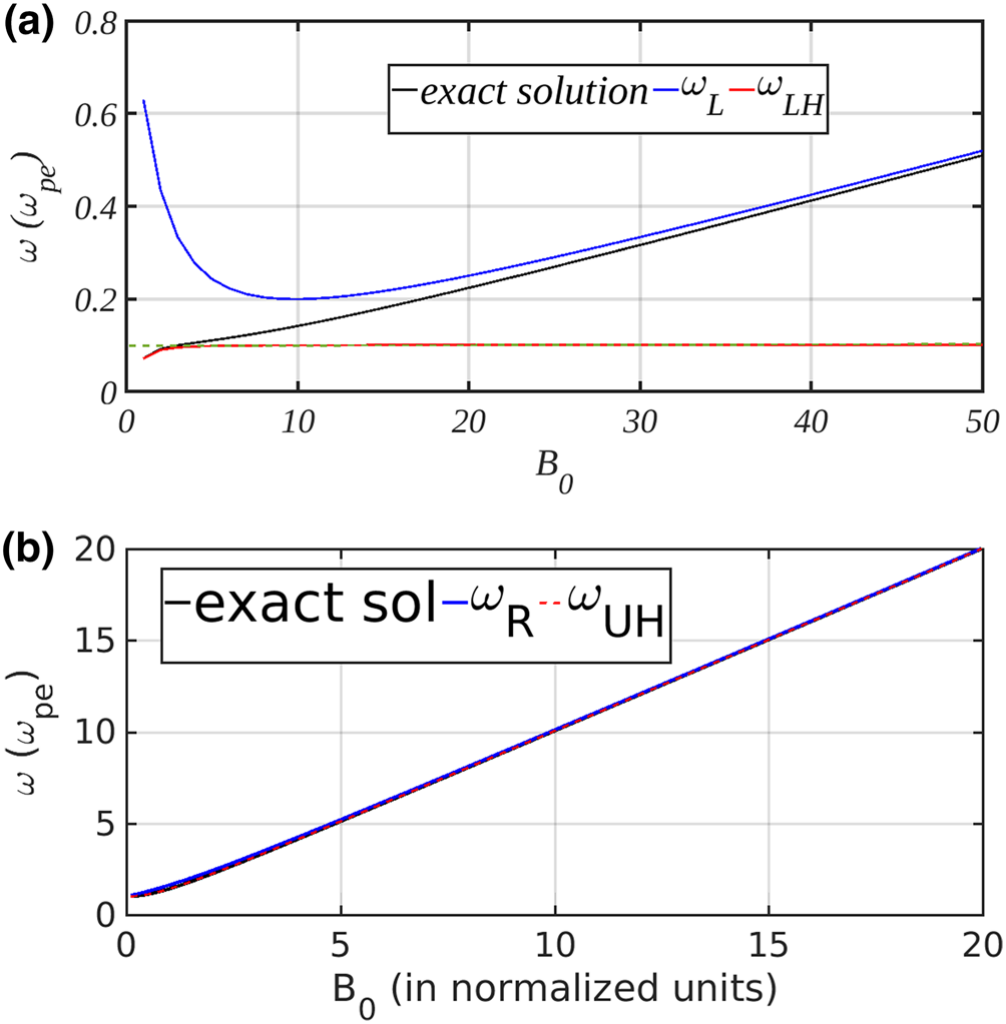
(**A**) Comparison of lower exact solution for $\omega$ (Eq. 6) with cut off frequency and analytical expression of LH as function of $B_0$. As one can observe that at lower magnetic field value, there’s gap between values of $\omega _{L}$ and $\omega _{LH}$ but at high value of magnetic field where exact solution of $\omega$ will play a role, it matches with $\omega _{L}$; signifying the shrink of stop band. Green dash-line indicates the $\omega _{pi}$ position in the plot. (**B**) Comparison of upper exact solution for $\omega$ with cut off frequency and analytical expression of UH as function of $B_0$. As one can observe that at lower magnetic field value there’s gap between values of $\omega _{R}$ and $\omega _{UH}$ but at high value of magnetic field where exact solution of $\omega$ will play a role it matches with $\omega _{R}$ indicative to the vanishing of stop band.

One comment should be made here about another solution in the upper end of frequency scale, it was found that at high magnetic field region $\omega _{ce}$ dominates all modes and cut off points and hence they merge very well. Plot of exact solution of Eq. (6) as function of $B_0$ applied is given in Fig. 4. As one can observe here gap between $\omega _R$ and $\omega _{UH}$ is very thin and at high magnetic fields they also merge indicating that stop band at upper frequency also vanishes with application of strong magnetic field.

### $\omega$ and ${k}$ analysis

In any dispersive medium, as the refractive index of media change spatially, the frequency of the EM wave remains same while its wavelength suffers a change. In this section we calculate the modified *k* and the phase velocity of incident laser pulse. We observe that by varying ambient magnetic field, phase velocity of laser pulse also changes (approaches velocity of light in vacuum, *c*) while decreasing the perturbations in the plasma.

**Figure 5:**
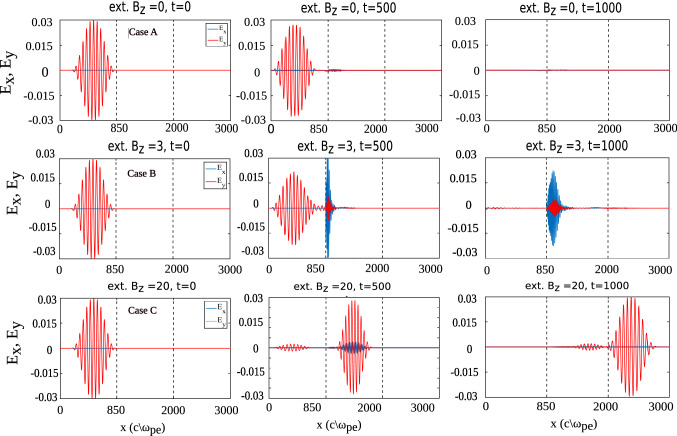
Spatial variation of $E_x$ and $E_y$ for the three cases at different times. Initially, only $E_y$ (due to laser field) is present in the system. In case (**A**), laser gets reflected from the plasma boundary without being able to interact with it. On the other hand, laser is able to interact with plasma in case (**B**) and we observe generation of $E_x$ in the system. In case (**C**), medium becomes transparent to laser and laser just passes through the plasma medium unhindered.

**Figure 6:**
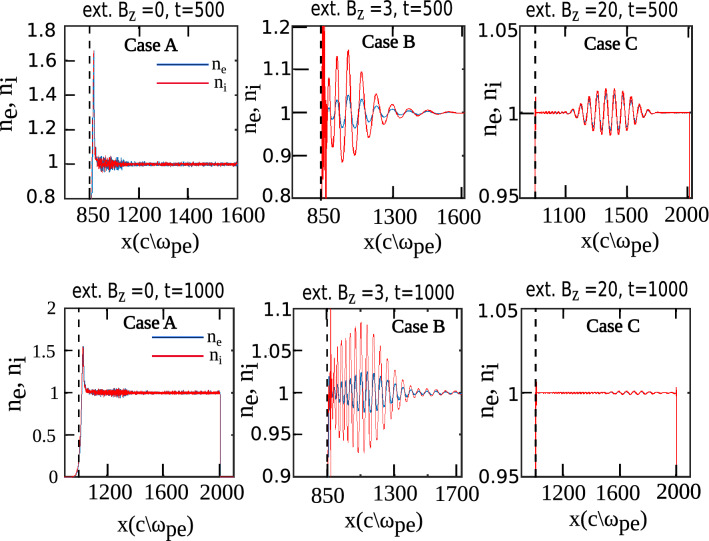
Spatial variation of number density of electrons and ions for the three cases at different times showing that plasma density gets perturbed at the interface for case (**A**) whereas for case (**B**), we observe density fluctuations in the bulk plasma as well which retain themselves even after laser has moved out of the simulation box ($t=1000$). For case (**C**), on the other hand, we observe some density perturbations in the bulk plasma at the time of interaction with laser ($t=500$) but these fluctuations are not retained by plasma after the interaction with laser is over.

Figure 5 shows a comparison of all three cases lying in Region I. Initially (at t = 0), electric field due to laser is present in the system. In case (A) ($B_0=0$), the laser interacts with plasma and gets reflected back from the plasma surface. However, for case (B) ($B_0 = 3$, satisfying the condition $\omega _{ce}> \omega _{l} > \omega _{ci}$), there are certain modes generated in plasma and as a result we observe a finite magnitude of $E_x$ in the system ^1,2^. $E_x$ that get generated in plasma have higher magnitude than $E_y$. On the other hand, in case (C) ($B_0=20$, satisfying the condition $\omega _{ce}> \omega _{ci} > \omega _{l}$), the plasma seems to be completely undisturbed by the laser as pulse freely propagates inside it without creating any perturbations in the medium and goes into vacuum space in the right side. The transparency induced in plasma on applying external magnetic field is the key observation of this work. Plasma density plots show that in case (A), plasma density at the interface is modified, on the contrary, for case (B), density perturbations are present in the bulk plasma as well. Ion density fluctuates more than electrons which propagates in longitudinal direction as can be seen at later times in Fig. 6. For case (C), there being density perturbations that can be seen at $t =500$ is due to laser field i.e. electrons and ions fluctuates with same amplitude, justifying our observation that  laser remains undisturbed via interaction with plasma in this case (Fig. 6).

**Figure 7:**
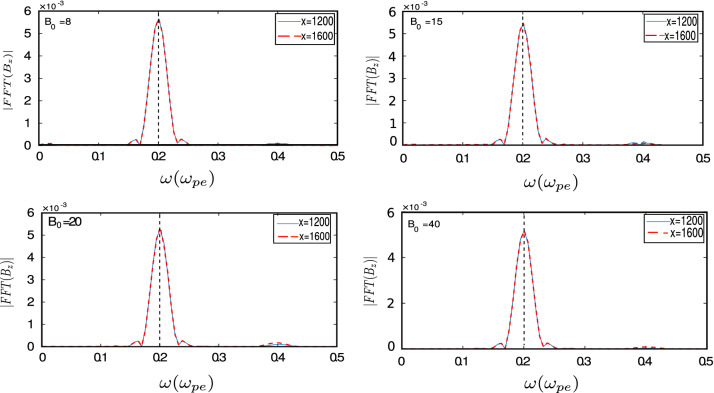
Fast Fourier Transform (FFT) of $B_{z}$ with time at two different locations in bulk plasma, showing that the peak frequency of electromagnetic wave does not change and it propagates unhindered in the plasma medium for different magnetic fields ($B_0=8, 15, 20, 40$).

**Figure 8:**
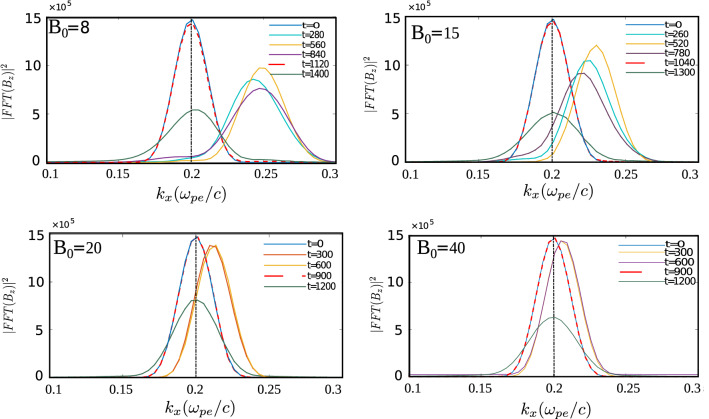
FFT of $B_{z}$ with x at different times showing that as laser interacts with plasma, $k_x$ shifts to a larger value than the initial $k_x$. This shift reduces as we increase the magnitude of applied magnetic field (shown in different subplots). This shift in $k_x$ is also reflected in velocity of EM wave approaching c in the medium (Table 4) and the transmission coefficient approaching unity (Table 3).

Figure 7 shows the Fast Fourier Transform (FFT) of $B_z$ of laser with respect to time for four different value of magnetic field, where transparency has been induced. It can be seen that the frequency of laser ($\omega _l=0.2$) does not change while propagating inside plasma (we show FFT of $B_z$ with time at two different values of x in the bulk plasma and obtain the same peak). However, $k_x$ of EM wave gets modified on propagation inside plasma (Fig. 8). The shift from the initial value of $k_x$ decreases on increasing applied magnetic field. We calculate the modified velocity of EM wave in plasma by peak frequency of the wave from the FFT and modified $k_x$ value (method II in Table 4) and found that velocity of the wave inside plasma approaches to *c* on increasing applied magnetic field (Table 4). In Table 4, we calculate velocity by two methods. In method I, we choose a point on the waveform and calculate the time taken by that point to cover a particular distance and method II includes calculation of velocity by modification in $k_x$. So we conclude that strong magnetization can stop pulse modification while pulse waveform is propagating through plasma media.

**Table 4:** Velocity of EM wave (normalized to c) in plasma with changing magnetic field.

$B_0$	Velocity from method I	Modified $k_x$ ($\omega _{pe}/c$)	Velocity from method II
8	0.78	0.2518	0.79
15	0.87	0.228	0.88
20	0.92	0.2137	0.94
40	0.96	0.2030	0.98

### Reversible and irreversible exchange of energy

In this study, we observed that depending on region and criteria, the laser energy exchange is either reversible or irreversible. As one can notice from Table 3, in region I criteria I there is significant absorption and this region is well explored in ref^1^. From these studies we know that in region I criteria I energy is dominantly coupled to ions and this coupling process is irreversible. While in region I criteria II, due to transparency, energy transfer is observe to be reversible. As when laser is present in the plasma, electrons and ions oscillate due to oscillating electric field and when the field passes through, they come to rest. In region II criteria I, laser reflects back due to formation of shielding fields so there’s no exchange of energy altogether. On the other hand region II criteria II is effectively Region I criteria II so there’s similar exchange of energy which is reversible.

In region III criteria I, we observe $97 \%$ transparency and reversible exchange of energy. This is quite different from other two regions, the reason behind this is simple. As the time scales of region III are same as electrons, with $B_0=3$ electrons are strongly magnetized. So that’s why laser is not able to couple its energy into electron effectively. To couple laser energy into electron irreversibly one has to weakly magnetize the electron and that can be achieved by ensuring another inequality i.e. $\omega _{ce}<\omega _{l}<\omega _{L}$. When we simulated with this condition by taking $B_0=0.25$, we observe $10.2\%$ absorption into electrons and about $2.2\%$ energy to ions irreversibly while $88 \%$ of laser pulse was reflected back.

Therefore, when the species are tightly bound to external magnetic field, they are not able to take energy from EM pulse irreversibly. That’s why in region I and criteria II when both the species were tightly magnetized to external magnetic field they were unable to couple their motion to laser pulse and that’s how pulse was transparent in this medium. In region III where it is in propagating region when electrons were tightly bounded we observe transparency for similar reason. One can argue that ions are not magnetized in this condition but this region’s time scales are fast so only electron motion is important here.

Now we move on to demonstrate the effect of charge separation on irreversible energy coupling. Under the effect of oscillating electric field and external magnetic field, the longitudinal drift can be written by Eq. (7)

7 $$\begin{aligned} {\vec{V}}_{\vec {E} \times {\vec{B}}}(t) = \frac{ \omega _{cs}^2}{ \omega _{cs}^2 - \omega _{l}^2} \frac{ {\vec {E}}(t) \times {\vec{B}}}{B^2} \end{aligned}$$

**Figure 9:**
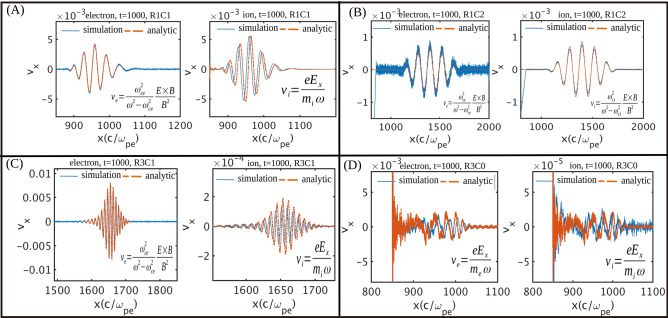
Comparison of electron and ion drift theoretically (Eq. 7) and simulation results. In criteria II both species are magnetized and their drift velocity in longitudinal direction is also same such that there’s no charge separation which can inhibit the propagation of EM wave (**B**). In the case when $\omega _l>\omega _{ce}$, there’s generation of electrostatic perturbation which helps in coupling laser energy into plasma irreversibly shown in subplot (**D**). For criteria I in region I and III, ions are un-magnetized while electrons are tightly bound to magnetic field [in subplot (**A**) and (**C**)].

Here, the suffix $s = e, i$ represents the electron and ion species respectively. In Criteria II, both ions and electrons are strongly magnetized and the dynamics are governed by the Lorentz force i.e. Eq. (7). As longitudinal velocity is independent of mass of the species for this criteria, there’s no possibility of charge separation such that there are no shielding fields to restrict the EM pulse propagation. A comparative analysis has been done between analytical drift given in Eq. (7) and numerical longitudinal drift experienced by both ion and electron in subplot (B) of Fig. 9 and it can be observed that there’s no velocity difference between species which results in no net charge density separation and hence EM wave is able to propagate unhindered. In Criteria I, electrons follow Eq. (7) while ion motion is governed by electric fields as they are unmagnetized. This is shown in subplot (A) and (C) of Fig. 9. We observe that they match well. When $\omega _{ce}<\omega _{l}$ (named as Criteria 0), both species are unmagnetized and they follow the longitudinal electric field (see subplot (D) of Fig. 9).

For a finite electromagnetic pulse the plasma species (electron and ions) also experience the ponderomotive pressure. The difference between the ponderomotive force experienced by electron and ions can lead to electrostatic excitations. However, it has been shown in ref.^36^ that in the X-mode configuration the ponderomotive pressure is same for ions and electrons at very strong magnetic fields. Thus, a finite EM pulse can also propagate undisturbed.

## Conclusion

A detail PIC simulation has been carried out by us to show complete transparency of EM wave radiation through a plasma in the presence of strong ambient field. The strength of the magnetic field has to be strong enough to elicit magnetized response from both electron and ion species at the EM wave frequency. The effect does not require relativistic intensity of the EM wave. This study finds it’s relevance in many fields of application where deposition and transfer of EM energy is required. This is achieved by appropriately tailoring the magnetic field arrangements to one’s desirability. Such special configured magnetic fields are useful in field of optics where pulse modulation is undesirable. This study can be of relevance in the plasma stealth technology. More rigorous studies are required in this regard to comment any further. We feel that these observations will have important significance in the context of astrophysical plasma near pulsar and magnetars where the magnetic field is quite strong and would elicit magnetized plasma response for typical EM frequencies of interest. In conclusion, our study proves that, under external magnetic effects, plasma can lose its collective behaviour.
